# Exaptation Traits for Megafaunal Mutualisms as a Factor in Plant Domestication

**DOI:** 10.3389/fpls.2021.649394

**Published:** 2021-03-24

**Authors:** Robert N. Spengler, Michael Petraglia, Patrick Roberts, Kseniia Ashastina, Logan Kistler, Natalie G. Mueller, Nicole Boivin

**Affiliations:** ^1^Department of Archaeology, Max Planck Institute for the Science of Human History, Jena, Germany; ^2^Department of Anthropology, Smithsonian Institution, National Museum of Natural History, Washington, DC, United States; ^3^School of Social Science, The University of Queensland, Brisbane, QLD, Australia; ^4^Department of Archaeology, Washington University in St. Louis, St. Louis, MO, United States; ^5^Department of Anthropology and Archaeology, University of Calgary, Calgary, AB, Canada

**Keywords:** ecosystem engineering, megafauna, crops, seed dispersal, endozoochory, domestication, origins of agriculture, exaptation

## Abstract

Megafaunal extinctions are recurring events that cause evolutionary ripples, as cascades of secondary extinctions and shifting selective pressures reshape ecosystems. Megafaunal browsers and grazers are major ecosystem engineers, they: keep woody vegetation suppressed; are nitrogen cyclers; and serve as seed dispersers. Most angiosperms possess sets of physiological traits that allow for the fixation of mutualisms with megafauna; some of these traits appear to serve as exaptation (preadaptation) features for farming. As an easily recognized example, fleshy fruits are, an exaptation to agriculture, as they evolved to recruit a non-human disperser. We hypothesize that the traits of rapid annual growth, self-compatibility, heavy investment in reproduction, high plasticity (wide reaction norms), and rapid evolvability were part of an adaptive syndrome for megafaunal seed dispersal. We review the evolutionary importance that megafauna had for crop and weed progenitors and discuss possible ramifications of their extinction on: (1) seed dispersal; (2) population dynamics; and (3) habitat loss. Humans replaced some of the ecological services that had been lost as a result of late Quaternary extinctions and drove rapid evolutionary change resulting in domestication.

## Introduction

All plants rely, to varying degrees, on ecosystem services performed by animals, including nitrogen transport, carbon respiration, topsoil reworking, removal of competitive vegetation, controlling of pests, decomposition of organic material, and dispersal of seeds and pollen. Many of these services, especially for seed and pollen dispersal, are so advantageous that plants evolve energetically costly traits to recruit animals into mutualistic relationships (Tiffney, 1981, 1984, 2004; Friis and Crepet, 1987; Wing and Tiffney, 1987; Meredith et al., 2011). Angiosperms are particularly adept at developing such mutualisms, and essentially all domesticated plants are angiosperms (Tiffney, 2004; Eriksson, 2008). The evolution of early domestication traits in plants is an example of the recruitment of humans for ecological services, notably seed dispersal (Spengler, 2020). Animals cyclically evolve into larger lifeforms, which fill a keystone ecological niche, but are highly susceptible to population collapse and extinction (Ferretti, 2008; Froyd et al., 2014; Estes et al., 2016; Malhi et al., 2016). These repeated megafaunal (>40 kg, as a rather arbitrary cutoff commonly cited in the literature) evolutions and subsequent extinctions have affected angiosperm evolution. The late Pleistocene (126,000–12,000 years ago) megafaunal extinctions also applied different evolutionary pressures on the plants that relied on zoochory; in some cases, these adaptations involved the recruitment of new dispersers, notably humans. In this article, we suggest that some of the plant traits for long-distance endozoochoric dispersal, such as rapid annual growth, self-compatibility, heavy investment in reproduction, high plasticity, and rapid evolvability, provided exaptations for agriculture.

**Figure 1:**
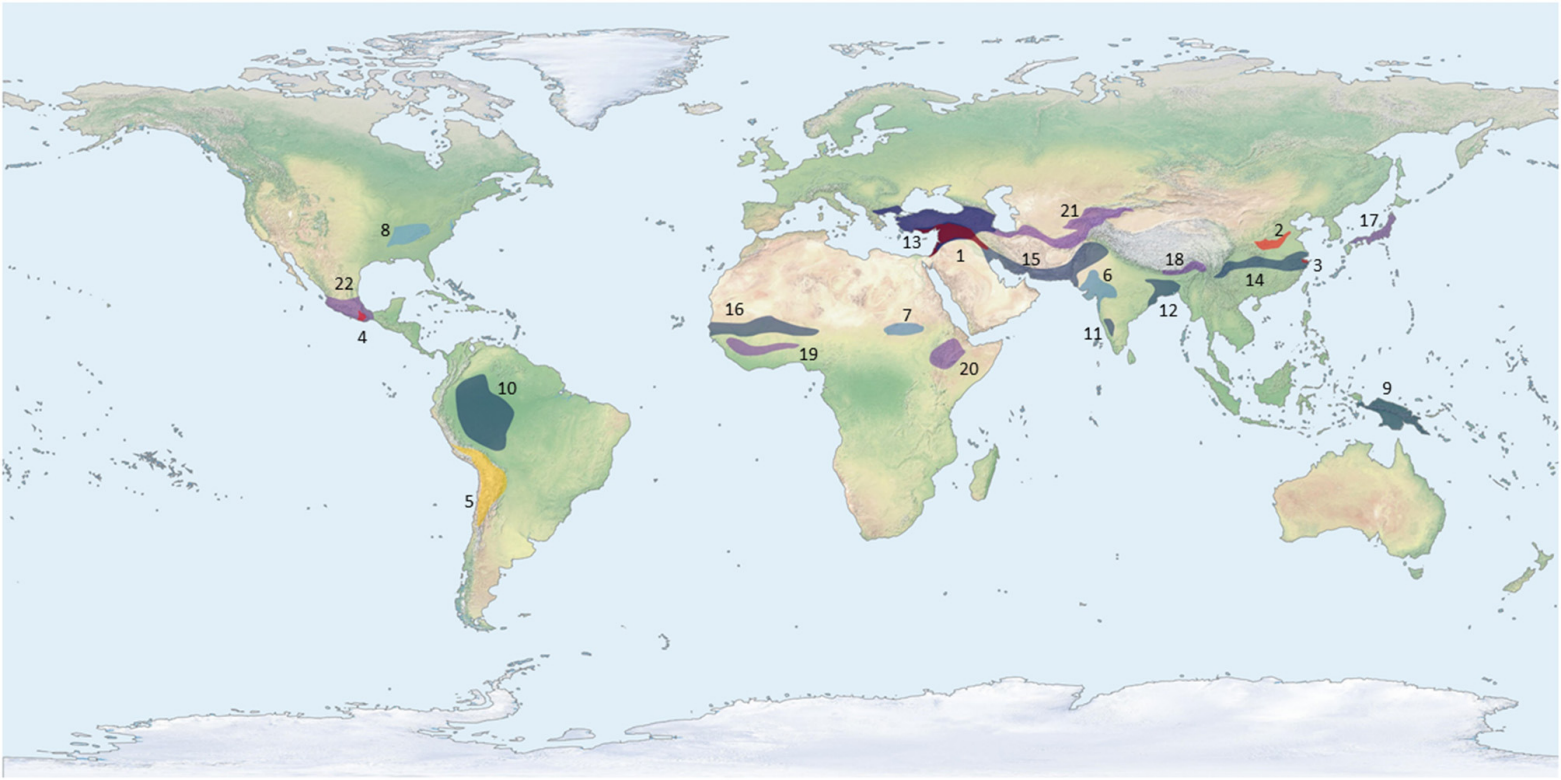
Map showing centers or regions of crop domestication. Each color corresponds to a different millennium, specifically indicating when the earliest trait of domestication was fully introgressed into the crop population for that center, a full chronology of these domestications is presented in Table 1. Only crops with a clear archaeobotanical signal for domestication are presented hear and the least controversial dates are presented. This map intentionally does not account for any concept of pristine or secondary centers.

The two main categories of endozoochoric (dispersed through animal ingestion) plants include: (1) those that produce fleshy fruits (Janzen and Martin, 1982) and (2) annual herbaceous plants with small seeds (Janzen, 1984). Most of the first traits of domestication in plants during the early or mid-Holocene were associated with seed dispersal *via* one of these two categories (Rindos, 1984; Spengler, 2020; Figure 1; Table 1). Given that, these traits allowed for mutualisms in the wild, their importance under cultivation implies an exaptation. We define exaptation following Gould and Vrba's (1982:4) original usage, as: “features that now enhance fitness but were not built by natural selection for their current role.” The use of exaptation, as opposed to preadaptation, avoids teleological pitfalls. We also recognize that an argument could be made for humans, as megafauna, being part of a broader adaptive guild, in which case the traits described here would not be exaptations, but simply adapted to earlier iterations of megafaunal mutualisms. Stepping away from the semantics or long-standing adaptationist debates, the key point remains pertinent, that many of the most important traits in plants for agriculture, evolved over the past 50 million years as parts of a series of systems for mutualisms with megafauna. Some of these physiological features, such as sugary fruits, are easy to visualize as exaptation traits allowing for agriculture. However, small-seeded annual crops have traits for survival in an anthropogenic ecosystem that were previously associated with non-human endozoochoric dispersal (Janzen, 1984; Kuznar, 1993; Spengler and Mueller, 2019). These traits include a lack of secondary defensive compounds, rapid annual growth, lack of defensive structures, dry small non-dehiscent fruits displayed on top of the plant, seeds smaller than 2.0 mm, hard protective seed coats, rapid evolvability, high developmental plasticity, and tolerance to trampling and readily disturbed environments. Domesticated plants with fleshy fruits, including many trees, cucurbits, and *Solanum* species, evolved larger fruits, higher sugar concentrations, thicker pericarp tissue, and often secondary traits, such as a reduction in alkaloids, protective structures, and increased size or architecture (Purugganan and Fuller, 2009; Fuller, 2018; Spengler, 2019). These suites of traits illustrate parallel evolutionary responses between: (1) the advantage of zoochory in the wild and (2) anthropogenic selective pressures under early cultivation.

**Table 1:** A list of centers or regions of domestication by millennium and the crops that evolved domestication traits there, linked numerically to Figure 1.

	**Region/center**	**Crop vernacular**	**Crop binomial**	**Earliest**
(1)	Southwest Asia	Einkorn, Emmer, Barley, Lentil, Pea, Chickpea, Bitter Vetch, Grass Pea, Fava Bean, and Flax^a^	*Triticum monococcum* ssp. *monococcum; T. turgidum* ssp. *dicoccum, Hordeum vulgare, Lens culinaris, Pisum sativum, Cicer arietinum, Vicia ervilia, Lathyrus sativus, Vicia faba, Linum usitatissimum*	Eighth Mill BC
(2)	North China Plains	Broomcorn and Foxtail Millet	*Panicum miliaceum, Setaria italic*	Fifth Mill BC
(3)	Yangtze Basin	Melons, Rice, Peaches, Apricots	*Cucumis melo, O. sativa japonica, Prunus persica P. armeniaca*	Fifth Mill BC
(4)	Oxaca Valley	Maize, Beans, Squash, Pepper	*Zea mays, Cucurbita pepo, Capsicum annuum, Phaseolus vulgaris*	Fifth Mill BC
(5)	Central/South Andes	Beans, Quinoa, Amaranth	*Phaseolus vulgaris, Chenopodium quinoa, Amaranthus caudatus*	Fourth Mill BC
(6)	Savannahs of West India	Mung Beans, Little Millet, Sesame, Horse Gram, Kodo Millet	*Vigna radiate, Panicum sumatrense, Sesamum indicum, Macrotyloma uniflorum, Paspalum scrobiculatum*	Third Mill BC
(7)	East Africa Savannah	Sorghum, Bottle Gourds	*Sorghum bicolor, Lagenaria siceraria*	Third Mill BC
(8)	Eastern North American Plains	Sunflowers, Chenopods, Sumpweed, Erect Knotweed, Squash, Little Barley	*Helianthus annuus, Chenopodium berlandieri, Iva annua, Polygonum erectum, Cucurbita pepo, Hordeum pusillum*	Third Mill BC
(9)	Island Southeast Asia	Bananas	*Musa acuminate, M. balbisiana*	Second Mill BC
(10)	Lowlands South America	Squash, Chocolate, Guava, Peanut, Peach Palm	*Cucurbita moschata/maxima, Theobroma cacao, Psidium* sp., *Arachis hypogea, Bactris gasipaes*	Second Mill BC
(11)	South India	Millet and Mung Beans	*Brachiaria ramosa, Vigna radiata*	Second Mill BC
(12)	East Indian Plains	Cucurbits, Rice, Pigeon Peas	Cucurbits, *Cajanus cajan, O. sativa indica*	Second Mill BC
(13)	Eastern Mediterranean	Grapes, Olives, Oats, Spelt, Bread Wheat, Rye, Lupine, Common Vetch	*Vitis vinifera, Olea europaea, Avena sativa, Triticum aestivum* ssp. *spelta, T. aestivum, Secale cereal, Lupinus albus, Vicia sativa*	Second Mill BC
(14)	Central China	Peaches, Apricots, Persimmon, Cannabis, Soy Beans	*Prunus persica, P. armeniaca, Diospyros kaki, Cannabis sativa, Glycine max*	Second Mill BC
(15)	Southwest Asian Exchange	Jujube, Citrus, Date Palm	*Ziziphus jujube, Citrus* spp*., Phoenix dactylifera*	Second Mill BC
(16)	West Africa Sahel	Pearl Millet	*Pennisetum glaucum*	Second Mill BC
(17)	Japanese Islands	Adzuki, Soy Beans, Barnyard Millet	*Vigna angularis, Glycine max, Echinochloa esculenta*	First Mill BC
(18)	Southern Himalaya	Common and Tatary Buckwheat	*Fagopyrum esculentum, F. tataricum*	First Mill BC
(19)	West Africa	Fonio and Guinea Millet, African Rice, Cow Pea	*Digitaria exilis, Brachiaria deflexa, Oryza glaberrima, Vigna unguiculata*	First Mill BC
(20)	Ethiopian Plateau	Teff, Finger Millet, Peas, Oats	*Eragrostis tef, Eleusine coracana, Pisum abyssinicum, Avena abyssinica*	First Mill BC
(21)	Silk Road Mountain Belt	Apples, Walnuts, Pistachios, Almonds, Russian Olives	*Malus domestica/pumila, Juglans regia, Pistacia vera, Prunus dulcis, Elaeagnus angustifolia*	First Mill BC
(22)	Southern Meso-America	Lima Bean, Common Bean, Squash, Chili	*Phaseolus lunatus, Phaseolus vulgaris, Cucurbita pepo, Capsicum annuum*	First Mill BC

a *Much of this information comes from Larson et al. (2014)*.

*Note that we only present species with clear evidence for domestication, noting when the traits of domestication were significantly introgressed into the cultivated population. We have left out crops selected for vegetative parts, because currently there is a lack of reliable data on when they evolved domestication traits and there are few discussions of what the selective pressures would have been that drove domestication*.

Many crop progenitors are extinct or endangered today and in several cases, wild populations demonstrate low rates of gene flow, reflective of reduced ability to colonize and escape ecological constraints. Likewise, changing herbivory pressures throughout the Holocene altered the distribution of suitable growing habitats and may have increased competition pressure (Spengler and Mueller, 2019). Often progenitors are restricted to marginal ecologies today, such as river floodplains (Hawkes, 1969; Rindos, 1984). The ecology of seasonally disturbed river edges mimics the disturbed habitats of agricultural fields, and both mimic the disturbance regimes of heavily grazed savannah. Riparian ecologies provide refuge to species with high dormancy rates and limited ability to disperse through space; some plants are able to continue in these settings longer than in non-disturbed locations, where they will be out competed. Exaptation traits from high-herbivory landscapes that increase adaptation to anthropogenic landscapes include a suite of endozoochoric features, rapid evolvability, and developmental plasticity. Links between megafaunal extinctions and the origins of agriculture have already been showcased in the Broad Spectrum Revolution Theory as a factor in human behavioral changes (Stiner, 2001; Kennett and Winterhalder, 2006); however, this theory states that the extinction of large game forced humans to focus more on seeds and less rewarding food sources. Rather than looking at possible economic pressures associated with the megafaunal extinctions, we look at changes in evolutionary pressures in plants and shifting ecosystem dynamics. We argue that the ecological ramifications of the late Quaternary extinctions set the stage for domestication in certain endozoochoric plants (Rindos, 1984; Purugganan and Fuller, 2009; Kistler et al., 2015; Spengler, 2019, 2020).

## Lost Seed-Dispersal Services

Mutualistic relationships for seed dispersal select for traits that allow seeds to move their offspring away from the parent plant, reducing parent-offspring and sibling-sibling competition (Howe and Miriti, 2000; Wang and Smith, 2002; Schupp et al., 2010; Tarszisz et al., 2018). Seed dispersal also reduces density-dependent mortality, a process linked to the Janzen-Connell Hypothesis (Nathan and Muller-Landau, 2000; Wang and Smith, 2002; Santamaría et al., 2007), interspecific competition (Garant et al., 2007), inbreeding depression (Nathan and Muller-Landau, 2000; Jara-Guerrero et al., 2018), and it promotes gene flow, diversification, adaptive evolution, and overall fitness (Eriksson, 2008). Seed dispersal allows for the colonization of new areas (Escribano-Avila et al., 2014), and dispersal by animals can increase the chance of seed deposition at ecologically favorable sites, what ecologists call directed dispersal (Eriksson, 2008). Nitrogen and water-absorbing organic material in dung also provides an ideal nursery for germinating seeds (Traveset and Verdú, 2002; Fuzessy et al., 2016). Studies show that megafaunal frugivores have a greater ability to disperse seeds and from a wider breadth of species than smaller animals (Peres and Van Roosmalen, 2002; Wotton and Kelly, 2011; Pires et al., 2018). Additionally, these large animals have longer gut passage times, allowing seeds to be moved over greater distances (Ruxton and Schaefer, 2012; Wotton and Kelly, 2012). Seed dispersal is an important evolutionary process in plants, as it ensures gene flow and changes in allele frequencies (Mayr, 1963; Ellstrand, 2014). Therefore, it should not be surprising that so many angiosperms have evolved similar traits in parallel to recruit animal dispersers.

### Small-Seeded Grains and Legumes

Among the ungulates, Artiodactyla (especially Ruminantia) evolved to digest plant cellulose in a four-chambered stomach, using fermentation and regurgitation with secondary mastication. The Perissodactyla retained a monogastric system, relying on intestines for digestion and are, therefore, unable to digest tough vegetative material and more often target sugary fruits. The success of the ruminant digestive system is evident in the sudden radiation and rapid dispersal of the clade across the Northern Hemisphere at about 53 million years ago. Seed dispersal of seeds <2 mm in diameter by Artiodactyla is well-documented (Nathan and Muller-Landau, 2000; O'Farrill et al., 2013) and can readily be observed through modern farm animals. Starting in the Eocene (56–33.9 million years ago), angiosperms began to evolve in response to heavy megafaunal herbivory pressure. Increased phytolith concentrations within and around plant cells appear to have coevolved with flat-crowned grinding teeth (Stebbins, 1981; Jacobs et al., 1999). The evolution of diminished lifecycles (annuals), rapid growth habits, and reduced floral and vegetative plant features, most pronounced in the florets of Poaceae or Cyperaceae, were likely responses to heavy herbivory (Vicentini et al., 2008). Likewise, the basal meristem of all grasses is presumed to be an adaptation for heavy herbivory and being consumed. Annuals invest more energy into reproduction, express reduced rates of inbreeding depression, and are prone to self-compatibility (Friedman, 2020); scholars have suggested that these traits may be linked to colonization and long-distance seed dispersal (Stebbins, 1950; Baker, 1972, 1965). In this way, some of the widely shared traits that evolved in parallel along with annual lifecycles appear to be tied to endozoochory, as a seed-dispersal syndrome. The world's first grasslands developed in the Northern Hemisphere in unison with herbivore ecosystem engineering, expressing mutualistic linkages between grazers and annuals (Jacobs et al., 1999). Following this, scholars have argued that herbivory with high-crowned dentition in grazing animals drove the expansion of grasslands and favored rapid-growing annual plants (Kaiser et al., 2013). Megafaunal extinctions of the Pleistocene appear to have led to denser vegetation cover and reduced diversity on some landscapes (Johnson, 2009; Bakker et al., 2016; Malhi et al., 2016) and forest loss on others (Escribano-Avila et al., 2014). Aggregate grazers can cause grasslands to grow faster and change community composition (Frank et al., 1998; Knapp et al., 1999; Geremia et al., 2019). While the fossil record appears to suggest the evolution of annuals prior to the Eocene, all of the most important clades of plants for agriculture evolved into their present forms during, arguably through coevolutionary linkages with megafauna, relatively recently in time.

**Figure 2:**
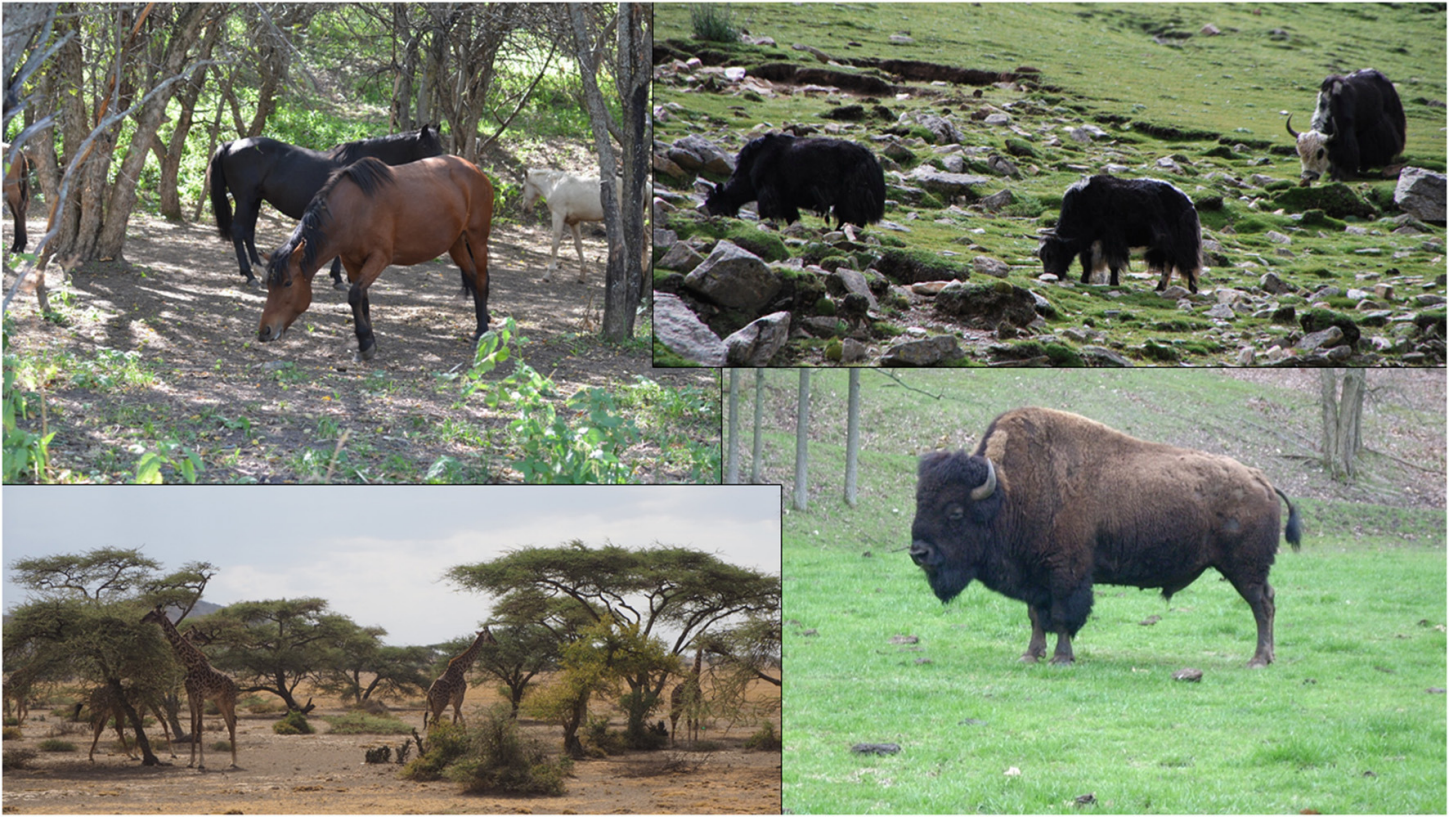
From top left: (1) Photo of horses consuming wild apples in the Tien Shan apple forests of Kazakhstan, photo taken by Artur Stroscherer and Martin Stuchtey. (2) Yaks grazing in Tibet have significant ecological impacts, photo by lead author. (3) Giraffes near the Olduvai Camp in Tanzania browsing on Acacia trees, photo taken by Yiming Wang. (4) Bison in Missouri on a heavily grazed field of herbaceous annuals, most of which are well adapted to heavy herbivory and disturbed ecosystems, photo by lead author.

Janzen (1984) first presented the theory of “Foliage as Fruit,” suggesting that these small annual plants evolved to be consumed by ruminant grazers as a way to disperse their seeds. Many of the plants in the grasslands of the early Holocene, when the first seed foraging humans crossed the Northern Hemisphere, had specific adaptations to a heavy herbivory landscape. Notably, they display seeds above terminal foliage and lack secondary metabolites or physical (thorns/spikes) defenses, making them the ideal food for ungulate grazers. Their seeds have hard testae, they remain small enough to pass through a ruminant digestive system, and have high dormancy rates (Spengler and Mueller, 2019). Some of these phenotypic traits are the first traits to change once humans take over as the main dispersers (Janzen, 1984; Kuznar, 1993; Spengler and Mueller, 2019). Many crop progenitors and ruderal or agricultural weeds express traits of endozoochoric dispersal and may have evolved on heavily grazed landscapes. The prominence of endozoochoric seeds in the stomach contents of frozen tundra Pleistocene grazers, studies of seed germination rates post-digestion in extant grazers, and rangeland composition studies all demonstrate the important role that megafauna provided to many progenitors (Spengler and Mueller, 2019) (Figure 2).

### Large Fleshy Fruiting Plants

Monogastic megafauna with browsing feeding behaviors readily disperse large seeds from within fleshy fruits. Extinction rates of Perissodactyla were much higher during the terminal Pleistocene. Despite high seed loss from mastication and digestion, the evolution of energetically costly sugary pericarp tissue and hard testae is testament to the adaptive advantage of endozoochory. Often the seeds within these fruits will not germinate without scarification or removal of the fruit coat; both are achieved through digestion (Traveset, 1998; Samuels and Levey, 2005; Robertson et al., 2006; Traveset et al., 2007). High dormancy rates in endozoochoric plants also prevent seed germination unless the seed has been consumed and dispersed (Baskin and Baskin, 2004; Tarszisz et al., 2018). In some cases, plants are able to maintain low rates of dispersal despite the loss of one or several dispersers (Guimarães et al., 2008; Jaroszewicz, 2013). Escribano-Avila et al. (2014) suggest that guilds of megafaunal browsers are responsible for woodland expansion in tropical South America. Jara-Guerrero et al. (2018) demonstrate that many large-fruiting trees in Central and South America are still dispersed by the few remaining megafauna, but that the general ability for dispersal has been greatly reduced. Studies of isolated populations of *Euterpe edulis* in the Brazilian Atlantic forest, illustrate that the functional extinction of dispersers can drive astonishingly rapid genetic changes in fruit size (Galetti et al., 2013).

Their ability to consume large amounts of fruits with large seeds make large-bodied animals particularly effective seed dispersers (Brodie et al., 2009; Campos-Arceiz and Blake, 2011; Sekar et al., 2015). Most megafruits are too large to be effectively disperse by medium-sized animals, which often function as seed predators (Guimarães et al., 2008; Jansen et al., 2012; Pires et al., 2014). In this paper, we define megafruit as any fleshy fruit too large for an avian disperser; in the temperate zones this is usually >25 mm, as Corvids are the largest avian dispersers, but in tropical zones with parrots and their kin, a cutoff of >40 mm has been posed (Onstein et al., 2018). Seed dispersal studies of small or medium-sized mammals and megafruits rather consistently demonstrate seed predation or weak dispersal ability (Murphy, 2018). In tropical zones, the guild of megafuanal dispersers is not restricted to mammals, and during the past few million years has included large-bodied birds and reptiles, notably tortoises (Valido and Olesen, 2007; Falcón et al., 2020). Studies of megafruit dispersal by large-bodied animals have been conducted in ecosystems around the world, including savannas and drier forest patches (Campos-Arceiz et al., 2008; Sekar and Sukumar, 2013; Sridhara et al., 2016) and in tropical forests (Kitamura et al., 2007; Harich et al., 2016). Several recent studies have shown that the megafaunal extinctions caused a loss or complete termination of seed-dispersal processes in many arborescent plants (Guimarães et al., 2008; Galetti et al., 2017; McConkey et al., 2018; Onstein et al., 2018; Pires et al., 2018; Lim et al., 2020).

Onstein et al. (2018) link megafaunal extinctions to an evolutionary decrease in the size of the fruits produced across the Arecaceae family and high rates of species extinctions throughout the Holocene (Lim et al., 2020). Purugganan (2019) used date palms (*Phoenix* spp.) as a case study for domestication through hybridization, noting that isolated populations of palms were brought into contact by humans. Spengler (2019) suggested, using archaeobotanical data from across Europe, that the mid-Holocene distribution of wild apples was restricted to glacial refugia. Apples, as with many domesticated perennials, appear to have rapidly evolved through hybridization, as humans dispersed plants between distant populations (Hughes et al., 2007; Cornille et al., 2014). Large-fruiting *Prunus* trees in Asia have restricted ranges and, in effect, the smaller the fruit, the wider the range today. Some of these species reproduce largely asexually despite heavy investment in a diaspore. The progenitor for the peach is thought to have once being widely distributed across northern China, but is now presumed extinct (Kostina, 1971; Lu and Bartholomew, 2003; Weisskopf and Fuller, 2014). Yu et al. (2018) and Su et al. (2015) suggest that megafaunal primates may have been a key component in the evolution of large fleshy fruits during the late Miocene or Pliocene.

Another informative case study of the loss of a megafaunal seed disperser comes from the Madagascar mega-lemurs (e.g., *Megaladapis edwardsi*), which went extinct as recently as two millennia ago (Burney et al., 2004; Crowley et al., 2011; Federman et al., 2016). Two of these extinct species, *Archaeolemur majori* and *Pachylemur insignis*, have been described as seed dispersers (Godfrey et al., 2008), and several others are thought to have been largely frugivorous (Crowley et al., 2011). Crowley et al. (2011) provide a list of trees with fleshy fruits that were likely dispersed by these giant primates, which today are losing range and have no ability to colonize new areas without human assistance (also see Rapanarivo, 1999). Some biologists have also noted that the most threatened large-fruiting species have seeds that are too large for successful dispersal by extant lemurs, which have smaller bodies than their precursors (Godfrey et al., 2008; Federman et al., 2016). Genetic studies of some of the trees in these vanishing forests show that they express genetic differentiation and fragmentation into subpopulations (Voigt et al., 2009; Federman et al., 2016). The introduction of new invasive species and domesticated herbivores to Madagascar, including horses, may further serve as surrogate dispersers (Vavra et al., 2007). The observations made in Madagascar are not unique, the loss of frugivores has led to a corresponding decrease in large-fruiting arborescent species around the world (Peres and Palacios, 2007; Stoner et al., 2007; Terborgh et al., 2008; Beaune et al., 2013; Tarszisz et al., 2018; Lim et al., 2020). Likewise, “rewilding” exercises, the practice of introducing animals with similar ecosystem functions as those lost during late Pleistocene or Holocene extinction events, serve as field studies to test theories relating to seed-dispersal mutualisms (Donlan, 2005; Griffiths et al., 2011). Many other studies have suggested population fragmentation, genetic heterogeneity, and extinction in plants that produce large fleshy fruits in response to megafaunal extinctions (Eriksson, 2008; Malhi et al., 2016; Galetti et al., 2017; Pires et al., 2018; van Zonneveld et al., 2018).

## Loss of Herbivory and Disturbance Regimes

Some paleoecologists have suggested that the extinction of herbivores caused shifts in vegetation cover and species composition, with some areas moving into secondary succession and dense forest cover, while other woody species lost their ability to colonize, causing forests to retreat (Johnson, 2009; Malhi et al., 2016), shifting vegetation densities and composition (Guimarães et al., 2008; Geremia et al., 2019). The reshaping of ecosystem structure shifted selection pressures and changed which plant species were dominant (Rule et al., 2012; Bakker et al., 2016; Malhi et al., 2016). The late Pleistocene megafaunal extinctions also may have caused evolutionary ripples in surviving animal populations, as predator pressures and competition changed (Estes et al., 2011). Scholars have also suggested that herbivore extinctions caused reductions in available nitrogen, calcium, and other soil nutrients (Doughty et al., 2013) and changed atmospheric composition (Doughty et al., 2010; Smith et al., 2010). Understanding the domestication of plants requires a better understanding of the shifting ecological pressures plant communities faced during the early Holocene.

Paleoecologists suggest that the extirpation of megafaunal grazers increased natural fire regimes and changed vegetation communities (Burney et al., 2003; Rule et al., 2012; Codding et al., 2014; Kaplan et al., 2016). As just one of many grazing and rangeland ecology studies, the results of years of fenced exclosure research of Yellowstone's Northern Range (Council, 2002), ecologists state that elk (*Cervus canadensis*) cause extreme reductions in woody plant growth, suppressing the regeneration of forests. Studies show that heavy bison (*Bison bison*) grazing on the North American grasslands dramatically alters vegetation communities and can replace the ecosystem services currently implemented by park service officials through controlled burning (Geremia et al., 2019). Bison and other megafaunal ruminants of the Pleistocene are described as creators and maintainers of grasslands or mosaic landscapes (Feranec et al., 2011; Gill et al., 2013; Malhi et al., 2016; Lundgren et al., 2018). Similar observations have been made regarding the loss of megafauna in Australia being linked to fire-prone landscapes before prehistoric peoples started partaking in controlled burning (Rule et al., 2012; Codding et al., 2014; Van Der Kaars et al., 2017). Many paleoecologists argue that the terminal Pleistocene extinctions in eastern North America increased woody vegetation and dead biomass buildup leading to increased magnitudes and rates of wild fires (Robinson et al., 2005; Gill et al., 2009; Gill, 2014). During the Holocene, in many parts of the world, humans became key ecosystem engineers, creating grasslands and mosaic landscapes through burning (Codding et al., 2014; Kaplan et al., 2016). Effectively, humans have replaced the ecosystem engineering roles of large mammals by clearing forests, burning vegetation, and turning over soil, constructing landscapes favorable to a certain community of plants (Kaplan et al., 2016).

Many crop or weed progenitors favor anthropogenically disturbed soils around villages or in cultivated fields, the Dump Heap Hypothesis (Anderson, 1952; Sauer, 1952), paralleling the highly disturbed settings of a bison wallow or a mammoth run. Scholars have long recognized that progenitors of annual crops share similar characteristics with agricultural field weeds and likely evolved for similar disturbance habitats (Hawkes, 1969; Baker, 1974; Vigne, 2015). This suite of traits is variably referred to as weedy, anthropophilic, commensal, or prone to disturbed environments. Scholars have argued that the anthropophilic traits of certain plants brought them closer to human settlements or “dump heaps” —areas where humans cleared woody vegetation, removed slower growing herbaceous competition, increased soil nutrient levels, and eventually maintained these “camp followers” (Anderson, 1952). Anthropophilic species tend to: (1) express high rates of developmental plasticity; (2) be better adapted to disturbed or dynamic ecosystems; and (3) have greater genetic evolvability (Baker, 1974; Geiger et al., 2018). Interestingly, anthropophilic traits, such as rapid growth (at the expense of overall competitiveness with slower growing species), lack of secondary chemicals or defensive structures, high dormancy rates, high rates of developmental plasticity, greater adaptability, short generations, prolific generation production, and rapid rates of evolutionary change, are all traits that favor landscapes with heavy herbivory pressure.

Most ecologists agree that agricultural weeds and many progenitors have exaptation traits allowing for success under disturbance regimes (Adams and Baker, 1962; Baker, 1965, 1972; Clements et al., 2004). Roughly half of the weedy species of annuals are selfing, leading to populations that tend to be genetically homogenous within a population, but highly divergent between populations (Friedman, 2020)—laying the foundations for hybridization (Clements et al., 2004). Selfing also allows a colonizing seed or founder population to reproduce even if it is not in pollinator range of a compatible mate, helping reestablish after disturbance (Stebbins, 1950; Baker, 1965, 1972). Weed ecologists have noted that selfing and high rates of dormancy often work together as mechanisms for dispersal, either through time on readily disturbed environments or across space as with long-distance colonization. Weedy annuals also tend to express remarkable capacities to tolerate treading, including by automobiles, suggesting adaptation to heavily trodden areas (Baker, 1974).

The mystery of this broad clade of weedy annuals is how they express such strong exaptation, given that the “level and frequency of fluctuations experienced in cropping systems is seldom encountered in more natural ecosystems” (Clements et al., 2004). Some ecologists have suggested that European weeds evolved on Pleistocene tundras, and gradually transitioned into agricultural fields (Salisbury, 1942; Godwin, 1960); other scholars have favored the riverbank or Floodplains Theory (Anderson, 1952; Sauer, 1952; Adams and Baker, 1962; Baker, 1965; Rindos, 1984). Given the prominence of seeds from weedy annuals in the stomachs of frozen megafauna in tundra ecosystems, it seems likely that weedy annuals evolved the suite of traits discussed above on heavily grazed fields with herbivores. These herbivore-heavy ecosystems would have dominated Pleistocene Europe and continued in many areas until the advent of farming, at which point endozoochoric annuals could have evolved into agricultural weeds, in a few cases they could have continued to evolve into field crops. The feedback-driven dynamics of human-weed ecology favor similar conditions as those seen on pre-Holocene landscapes, ecological conditions that today are limited to very small pockets outside anthropogenic setting, such as river floodplains or fire-prone landscapes.

The debate continues over what drove the high level of adaptability and invasiveness in weedy species, with two groups of thought: (1) those who argue that these plants primarily express a high degree of developmental plasticity (Neve et al., 2009); and (2) those who argue that they are capable of rapid genetic change (Baucom and Holt, 2009). Baker (1965) campaigned the view of adaptiveness through highly flexible developmental systems. He was also quick to note that many of these plants do evolve genetic responses rapidly and that plasticity itself is genetically programmed—i.e., not mutually exclusive viewpoints (Neve et al., 2009). Over the past few decades, with more genomic research on agricultural weed populations, scholars have increasingly recognized the remarkable ability for rapid evolutionary change in these plants. These genetic changes occur in a cultivated field on the scale of decades, and are increasingly being recognized as common ecological phenomena, notably in perturbed anthropogenic ecosystems (Thompson, 1999; Palumbi, 2001; Clements et al., 2004). The genetic basis of weedy and invasive traits may be tied into a genetically programed exaptation, allowing both rapid evolution and developmental plasticity (Baucom and Holt, 2009; Stewart et al., 2009). We argue that, one plausible explanations for the evolution of these traits across a remarkably diverse guild of plants, representing dozens of unrelated families is the collective evolutionary pathway toward adaptation to disturbed landscapes, most notably heavily grazed fields.

## Plant Domestication

The earliest traits of domestication in plants are associated with changes in seed-dispersal mechanisms (Rindos, 1984); this is well-recognized in the switch from dispersal by brittle rachises in wild cereals to sickle harvesting and seed sowing by humans, causing an evolutionary shift to the tough-rachised allele in the cultivated population (Ladizinsky, 1979; Fuller and Allaby, 2009; Langlie et al., 2014; Larson et al., 2014; Li and Olsen, 2016; Jones et al., 2021). Most of the earliest plants to express evolutionary traits of domestication had seeds that were dispersed by animals in their wild forms (Spengler, 2020). These endozoochoric crop progenitors fall into two broad categories, plants with fleshy fruits and annual herbaceous plants with small seeds—in both cases, megafaunal mammals likely played a primary role as seed dispersers. In Figure 1, we present a revised version of the earliest centers of domestication map, whereas we do not separate primary or secondary centers, and instead, see domestication as the result of increasingly more intense interaction between humans and select plant species. Likewise, in Table 1, we present a summary of the earliest crops domesticated in each center, as illustrated by morphological changes seen in archaeobotanical remains (following data accumulated by Fuller et al., 2014; Larson et al., 2014; Spengler, 2020). Ultimately, agriculture is better thought of as a complex mutualism between humans and plants, whereas domestication is a set of evolutionary changes associated with that mutualism. Several factors influence the rates of these evolutionary changes, including the insularity of cultivated populations and the presence of exaptation traits.

Rindos (1984) posited that the reason the oak tree was never domesticated, despite intensive human harvesting of acorns on three continents over several millennia, is because of the continued successful dispersal by Sciuridae. The prominent seed dispersal rates and strong wind pollination result in a different population structure than most large fruit trees (Petit et al., 2003; Curtu et al., 2007). Extreme generation lengths may also have hindered domestication in *Quercus*, but many long-generation fruiting trees did evolve in response to human selective pressures during the mid- to late Holocene (Miller and Gross, 2011; Fuller, 2018). A similar argument could be made for avian dispersed fruits, which in most cases, maintained successful seed-dispersal regimes into the Holocene; tellingly, there are few examples of small berries evolving domestication traits prior to directed breeding and the intentional implementation of hybridization (except *Vitis vinifera*). Miller and Gross (2011) note that protracted models of domestication might be expected for arborescent crops, and Fuller (2018) argued for protracted models in some species, but this is not the case from even a cursory look at long-generation perennial crops. In addition to their long generations, clonal propagation further reduced the number of sexual cycles separating wild and domesticated forms (Zohary and Spiegel-Roy, 1975). It is clear that hybridization is a key factor in long-generation perennial evolution in the wild and under cultivation, especially for endozoochoric species. Self-incompatibility among long-generation perennials also facilitates greater rates of interspecific hybridization (Hamrick et al., 1992; Ellstrand et al., 1996; Petit et al., 2003; Ward et al., 2005; Curtu et al., 2007; Miller and Gross, 2011). Hamrick et al. (1992) claim that among woody plants, endozoochoric species tend to have greater diversity within a species or a population; although, they do not consider whether extinction of dispersers increases or decreases diversity. The loss of megafaunal dispersers left many of the progenitors of modern crops with lower levels of gene flow, fragmentary and isolated populations, and heterogeneous population structures.

Domestication through hybridization is largely the result of genetically isolated populations being brought together as humans crossed their former seed-dispersal barriers. For example, roughly 3,000 years ago, as humans dispersed dates (*Phoenix dactylifera*) out of North Africa, they hybridized with *P. theophrasti*, a previously isolated wild relative (Purugganan and Fuller, 2009; Flowers et al., 2019). Isolated populations of phoenix palms existed across South and southwest Asia, notably in Yunnan, northern India, and the Arabian Peninsula during the mid-Holocene. Other South Asian fruits may have experienced similar range contractions when humans began to disperse their seeds, including *Ziziphus* spp., and *Citrus* spp., both of which were domesticated through hybridization for isolated wild populations. Similarly, in Central America, avocado (*Persea americana*), papaya (*Carica papaya*), and white sapote (*Casimiroa edulis*) were all megafaunal dispersed in their progenitor habitats, as were *Annona* spp., cashew (*Anacardium occidentale*), and guava (*Psidium guajava*) in South America.

Recent research into the domestication of peach palms (*Bactris gasipaes*) in South America has identified a heterogeneous mosaic of wild relatives, which appear to have played varying roles in the domestication process (Clement et al., 2017). Clement et al. (2017:149) note that “dispersal by humans is effective for crossing barriers” and shifting distributions of Late Glacial Maximum populations of the palms are a key aspect of domestication. *Musa* spp. provides another interesting case study of perennials evolving through human-driven barrier crossing. Most cultivated bananas today are in the Eumusa section, and are diploid or triploid hybrids, involving *Musa acuminata* × *M. balbisiana*. Genetic studies show that hybridizations between different *M. acuminata* subspecies occurred in island Southeast Asia and western Melanesia, and involved humans dispersing the plants between islands (Perrier et al., 2011). This hybridization led to parthenocarpy and eventually fed into a selection for sweeter fleshier fruits; illustrating, yet again, that the earliest traits of domestication are tied into seed dispersal (Heslop-Harrison and Schwarzacher, 2007). Further hybridization led to the tetraploid forms (Ude et al., 2002; Raboin et al., 2005).

North American wild cucurbits may have had a much wider range of distribution in the mid-Holocene than today (Fritz, 1999)—currently rare or extinct. There are at least five domesticated species, each brought under cultivation in different regions of the Americas and at different times (Nee, 1990). Kistler et al. (2015) used high-throughput sequencing to analyze complete placid genomes of 91 ancient domesticated, modern domesticated, and modern wild specimens. Additionally, they studied bitter taste receptors in extant megafaunal mammals and note that the toxic and bitter triterpenoid compounds in wild cucurbits would have been less likely to deter large mammals than small mammals. They argue that the extinction of large-bodied seed dispersers across the northern portion of the Americas dramatically altered ecological pressures on wild populations and illustrated that population fragmentation was the direct result of the loss of megafaunal dispersers, and the domestication of all modern squashes is the result of humans taking on the role of surrogate megafaunal dispersers (Kistler et al., 2015).

### Exaptation Traits Supporting Domestication

**Figure 3:**
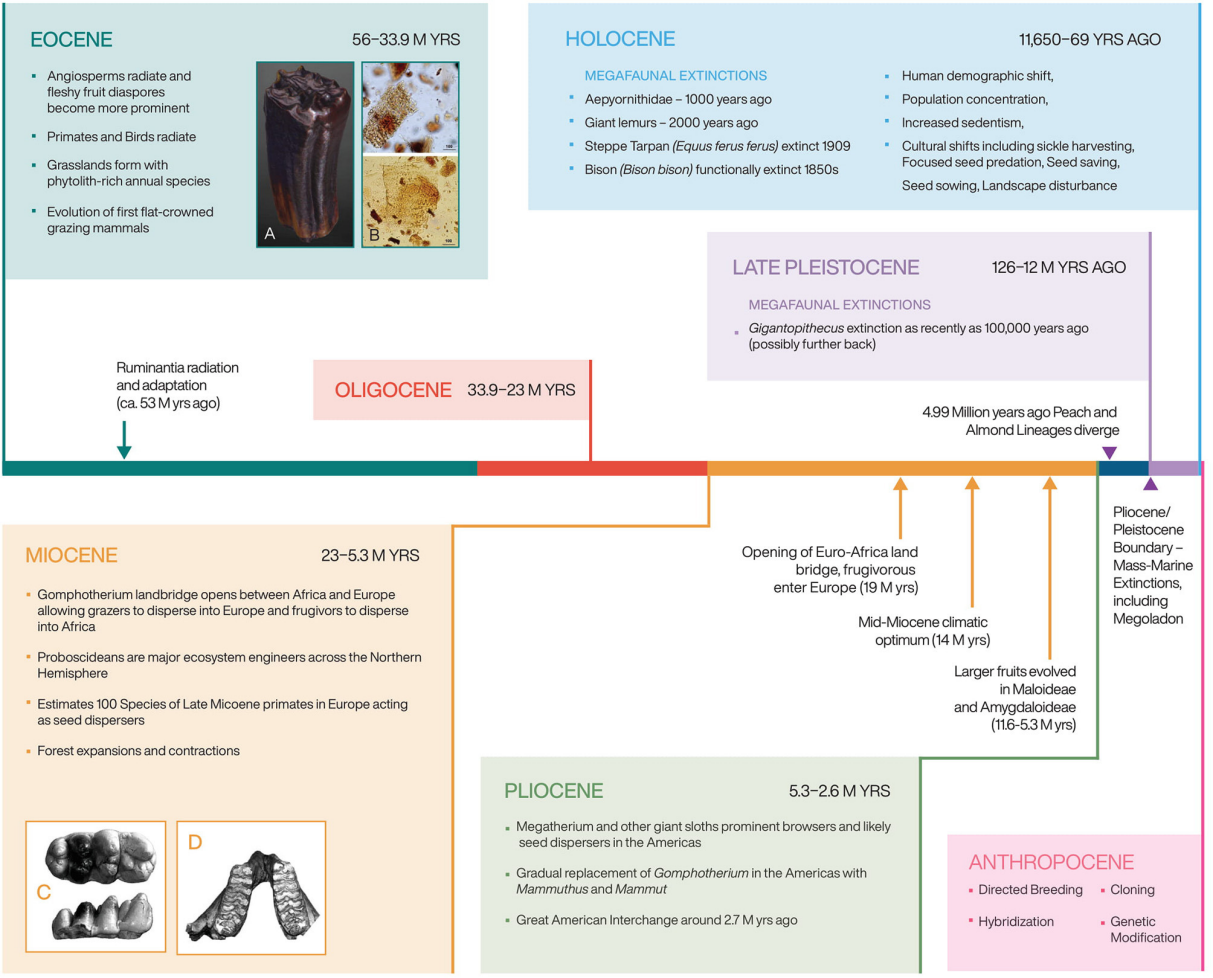
A timeline showing key coevolutionary processes that created mutualistic relationships between large mammals and crop and weed progenitors. A deep time look at domestication helps lay the foundation for understanding evolution under cultivation. Elephantine dental structure depictions from Ferretti (2008) and horse teeth provided by William Taylor.

Scholars have argued that the “key to successful domestication of a crop lies… in its genetic endowment,” claiming some plants express genetic traits that allow for domestication (Murphy, 2007). Scholars have also pointed out that the small-seeded annual crop progenitors, including most agricultural weeds, were “specifically pre-adapted to growth and competition for resources in agriculture-like environments” (Vigueira et al., 2013). They further state that “species that are naturally adapted to disturbed habitats may be pre-adapted to the agricultural environment” (Vigueira et al., 2013). It is not a coincidence that only grasses and Core Eudicots have proven suitable for cultivation; furthermore, within this grouping, only a handful of families have been adopted as field crops, including Poaceae, Amaranthacaeae, Fabaceae, Brassicaceae, Lamiaceae, Solanaceae, and Rosaceae. These families are all highly specious and, with the exception of the grasses, are all relatively recently evolved lineages. There is reason to argue that the speciation and diversification of these families occurred in part due to coevolutionary linkages with megafaunal mammals, mostly since the Eocene. We argue that many of the exaptation traits that allowed plants to become crops or weeds, evolved as adaptations to heavy herbivory, including rapid evolvability, developmental plasticity, and likelihood of change through hybridization. This argument would also imply that farming, as it exists today, could not have evolved on Earth during an earlier period (Figure 3).

Each interglacial forced the ancestral populations of domesticated plants to migrate across an entire continent (Clark, 1998). The remnant fragmentary populations of large-fruiting trees across: (1) North America, *Maclura pomifera, Asimina triloba*, and *Prunus Americana*; (2) Europe, *Sorbus domestica, Mespilus germanica, Pyrus pyraster*, and *Malus sylvestris*; (3) Asia, *Prunus mira, P. davidiana*, and *Diospyros kaki*; and (4) South America, *Annona cherimola, A. muricataspeak*, and *Eugenia stipitata*, speak to the ecological effects of the large mammalian extinctions (Janzen and Martin, 1982). Genetically isolated refugial populations often hybridize when brought into contact, expressing heterosis and in some cases polyploidy (Faurby and Svenning, 2015). There were rapid changes in allele frequencies in these populations once humans dispersed them across the melted glacial barriers (or between oases, islands, or isolated forest patches) during the mid-Holocene. Domestication occurs quicker on mosaic or fragmentary landscapes with lower levels of gene flow, following the basic principles of Island Biogeography (Rindos, 1984).

The idea that domestication in certain species arose from hybridization of glacial refugial or isolated forest populations has been suggested for several crops, including: apples (*Malus domestica/pumila*) (Cornille et al., 2014); bananas (polyploidy) (Heslop-Harrison and Schwarzacher, 2007); dates (*Phoenix dactylifera* × *P. theophrasti*; Flowers et al., 2019), (*P. dactylifera* × *P. canariensis*; (González-Pérez et al., 2004); cannabis (*Cannabis sativa*) (Clarke and Merlin, 2013; Small, 2015); citrus (Moore, 2001; Wu et al., 2014); cucurbits (Kistler et al., 2015); walnuts (*Juglans regia*) (Pollegioni et al., 2015); and wheat (polyploidy) (Marcussen et al., 2014). Hybridization can also introgress favorable traits into crops, such as non-shattering into *indica* rice (Gross and Zhao, 2014) or frost tolerance into Himalayan barley (Zeng et al., 2018), allowing for the expansion of crops into regions with novel ecological constraints (Pfennig et al., 2016; Janzen et al., 2019). Scholars have long recognized the important role of hybridization in plant evolution and domestication more specifically (Anderson, 1949; Stebbins, 1950; Anderson and Stebbins, 1954; Ellstrand et al., 1996; Arnold, 1997; Rieseberg and Carney, 1998; Hughes et al., 2007). The prominence of interspecific hybridization in plants often drives adaptive evolution, a process well-documented in early agricultural fields (Purugganan and Fuller, 2009; Hancock, 2012; Arnold, 2014). Perennials are disproportionately more likely to double their genome upon hybridization, due to the increased number of replication errors possible throughout one individual's lifetime (Friedman, 2020). Miller and Gross (2011) note that among fruit trees, most crops have multiple domestication events, often through hybridization, and in many cases resulting in multiple domesticated species. They single out the genera of *Annona, Artocarpus, Citrus, Diospyros*, and *Prunus*, as having multiple domesticated species and hybrid complexes. The switch from unisex to bisexual flowers is possibly linked to limited mate selection and self-incompatibility, especially on clonally propagated orchards (Miller and Gross, 2011). Ultimately, the isolated nature of many of the crop-progenitor populations facilitated hybridization and the expression of heterosis or polyploidy once humans began dispersing them.

## Anthropogenic Ecosystem Services

Megafaunal extinctions caused range reductions, genetic heterogeneity, and extinction of many crop progenitors. The loss of ecosystem services performed by megafaunal grazers and browsers set the stage for plant domestication in the early and mid-Holocene. It is also interesting to note that many domesticated animals represent megafauna that were approaching extinction when they developed mutualistic relationships with humans. Likewise, many field weeds and anthropophillic plants have traits that make them prone to disturbed environments, which include areas around human settlement and heavily grazed fields with large herd animals (Hawkes, 1969). The earliest traits of domestication in many crops are associated with endozoochoric dispersal (Janzen, 1984; Kuznar, 1993; Purugganan and Fuller, 2009; Spengler and Mueller, 2019). Ultimately, humans have served as the greatest “rewilding” force for the megafaunal anachronisms, simultaneously: (1) dispersing seeds of plants that had lost part or all of their ability for dispersal; (2) clearing and disturbing soils creating conditions favored by species that evolved on heavy herbivory landscapes; (3) maintaining fruiting tree species; and (4) causing hybridization between previously isolated plant lineages. When considering plant an animal domestication, it is essential to explore the evolutionary legacy that led up to the mutualistic relationships that humans engage in today.

The reframing of plant domestication as a set of traits evolving to support an mutualistic relationship is a growing perspective in the literature. As scholars, reframe the discussion, they can stop searching for rational drivers of human decision making, such as climate change or population pressure, and instead focus on the ecology and evolutionary history of the plants involved. Future discussions of these dispersal processes in the progenitors of modern crops would better be served by a more detailed understanding of the megafaunal mammals that existed in the regions where plants evolved domestication traits. Likewise, a clearer understanding of the digestion and preferences in food choices among extant representatives of these mammalian lineages would help pinpoint specific species or guilds linked in coevolutionary relationships with the respective plant clades. New applications of aDNA can better link ancient dispersers and their plants, as well as identifying the population legacies of disperser extinctions (e.g., Kistler et al., 2015). Future studies of plant domestication would greatly benefit from collaborations between ecologists, paleontologists, and archaeologists. Traditionally, these are disciplines that have not mingled and we would argue that the lack of crosspollination of ideas has hampered the development of domestication research. The traits of domestication seen in the archaeological record parallel evolutionary processes in the wild; therefore, a better understanding of the archaeological record could come from discussions with evolutionary ecologists. Likewise, many traits in plants that favored farming originally evolved on now extinct landscapes; therefore, the missing variables for understanding early plant domestication may come from restored megafaunal landscapes, such as the bison preserves of the American Midwest, Białowieski National Park in Poland, or Pleistocene Park in Sakha Republic, Russia (e.g., Mueller et al., 2020).

## Data Availability Statement

The original contributions presented in the study are included in the article/supplementary material, further inquiries can be directed to the corresponding author.

## Author Contributions

All authors listed have made a substantial, direct and intellectual contribution to the work, and approved it for publication.

## Conflict of Interest

The authors declare that the research was conducted in the absence of any commercial or financial relationships that could be construed as a potential conflict of interest.
